# Synthesis, crystal structure and charge-distribution validation of β-Na_4_Cu(MoO_4_)_3_ adopting the alluadite structure-type

**DOI:** 10.1107/S2056989016010367

**Published:** 2016-07-12

**Authors:** Wassim Dridi, Mohamed Faouzi Zid

**Affiliations:** a Laboratoire de Matériaux et Cristallochimie, Faculté des Sciences de Tunis, Université de Tunis El Manar, 2092 El Manar Tunis, Tunisia

**Keywords:** crystal structure, BVS, CHARDI, alluaudite-type, sodium, copper(II), molybdate(VI)

## Abstract

Single crystals of a new variety of tetra­sodium copper(II) tris­[molybdate(VI)], Na_4_Cu(MoO_4_)_3_, have been synthesized by solid-state reactions and characterized by single-crystal X-ray diffraction. This alluaudite structure-type is characterized by the presence of infinite layers of composition (Cu/Na)_2_Mo_3_O_14_ parallel to the (100) plane, which are linked by MoO_4_ tetra­hedra, forming a three-dimensional framework containing two types of hexa­gonal channels in which Na^+^ cations reside. The Cu^2+^ and Na^2+^ cations are located at the same general site with occupancies of 0.5. All atoms are on general positions except for one Mo, two Na (site symmetry 2) and another Na (site symmetry -1) atom. One O atom is split into two separate positions with occupancies of 0.5. The title compound is isotypic with Na_5_Sc(MoO_4_)_4_ and Na_3_In_2_As_3_O_12_. The structure model is supported by bond-valence-sum (BVS) and charge-distribution CHARDI methods. β-Na_4_Cu(MoO_4_)_3_ is compared and discussed with the K_4_Cu(MoO_4_)_3_ and α-Na_4_Cu(MoO_4_)_3_ structures.

## Abstract 2

A new variety of tetra­sodium copper(II) tris­[molybdate(VI)] is characterized by the presence of infinite layers of composition (Cu1/Na1)_2_Mo_3_O_14_ parallel to the (100) plane, which are linked by MoO_4_ tetra­hedra, forming a three-dimensional framework containing two types of hexa­gonal tunnels in which Na^+^ cations reside.

## Chemical context   

In recent years, a number of molybdates have received considerable attention due to their amazing properties and high application potential in various fields, such as photoluminescence (Shi *et al.*, 2014 ▸) and Li-ion batteries (Reddy *et al.*, 2013 ▸). For example, the copper molybdate Cu_3_Mo_2_O_9_ doped with lithium displays high Coulombic efficiency in lithium-ion batteries and excellent charge-discharge stability (Xia *et al.*, 2015 ▸). Many new molybdate phases have been synthesized and structurally characterized by X-ray diffraction, among which a large number belong to the alluaudite type, such as Na_25_Cs_8_Fe_5_(MoO_4_)_24_, which presents a high electrical conductivity (Savina *et al.*, 2014 ▸). The alluaudite-type structure was first determined on natural minerals by Fisher, who showed that alluaudite compounds crystallize in the monoclinic *C*2/*c* space group (Fisher, 1955 ▸). Moore proposed the general formula *X*(2)*X*(1)*M*(1)*M*(2)_2_(PO_4_)_3_, in which the *X* and *M* mono-, bi- or trivalent cations are written according to their size (*X* are large cations and *M* are distorted octahedrally coordinated atoms). It represents the parental structure-type of the group referred to as alluaudite-type (Moore, 1971 ▸). The size of the channel and the stability of the alluaudite network led to many phases belonging to this structural type. We can totally or partially replace not only the monovalent cations, but also the central atoms of the *M*O_6_ octa­hedra and *T*O_4_ tetra­hedra. It is also possible to make substitutions with cations in different oxidation states adopting the same type of coordination number (Mo^6+^, V^5+^, P^5+^ and As^5+^). Alluaudite molybdates usually have the general formula of *X*(2)*X*(1)*M*(1)*M*(2)_2_(MoO_4_)_3_ and adopt the *C*2/*c* space group, with *a* ≃ 12, *b* ≃ 13 and *c* ≃ 7 Å, examples being the K_0.13_Na_3.87_MgMo_3_O_12_ (Ennajeh *et al.*, 2015 ▸), Na_3_Fe_2_(MoO_4_)_3_ (Muessig *et al.*, 2003 ▸) and Na_4_Co(MoO_4_)_3_ (Nasri *et al.*, 2014 ▸) compounds. A review of the literature also reveals the presence of other formulae, such as Na_5_Sc(MoO_4_)_4_, Na_2_Ni(MoO_4_)_2_ (Klevtsova *et al.*, 1991 ▸) and Na_2.2_Zn_0.9_(MoO_4_)_2_ (Efremov *et al.*, 1975 ▸), which crystallize in the space group *C*2/*c* with cell parameters of about *a* ≃ 12, *b* ≃ 13 and *c* ≃ 7 Å. All alluaudite-type compounds can be described by the general formula given by Moore (1971 ▸), but their structures can differ by the number of formula units per unit cell. They are characterized by a three-dimensional heteropolyhedral frame­work formed by *T*O_4_ tetra­hedra and *M*O_6_ octa­hedra, delimiting two types of channels running along the *c* axis. A new variety of β-Na_4_Cu(MoO_4_)_3_ formulation was obtained by a reaction in the solid state at 873 K.

## Structural commentary   

**Figure 1:**
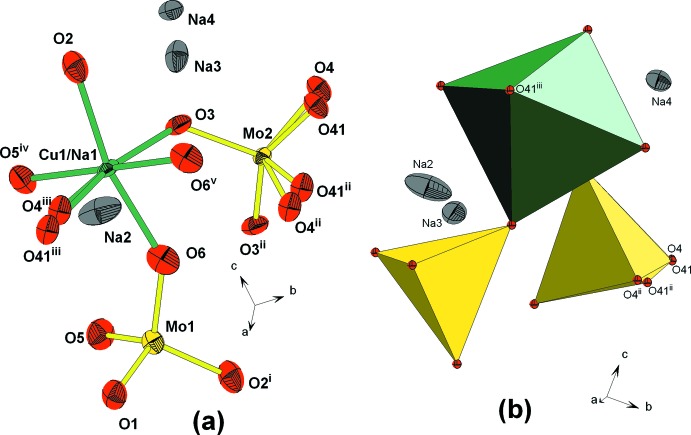
Representation of the coordination polyhedra in the structural unit of β-Na_4_Cu(MoO_4_)_3_, showing (*a*) full atomic, (*b*) polyhedral. All atoms are represented as displacement ellipsoids at the 50% probability level. [Symmetry codes: (i) *x*, *y*, *z* − 1; (ii) −*x* + 1, *y*, −*z* + 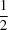; (iii) *x* + 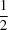, −*y* + 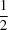, *z* + 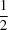; (iv) *x*, −*y*, *z* + 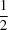; (v) −*x* + 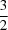, −*y* + 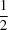, −*z* + 1.]

**Figure 2:**
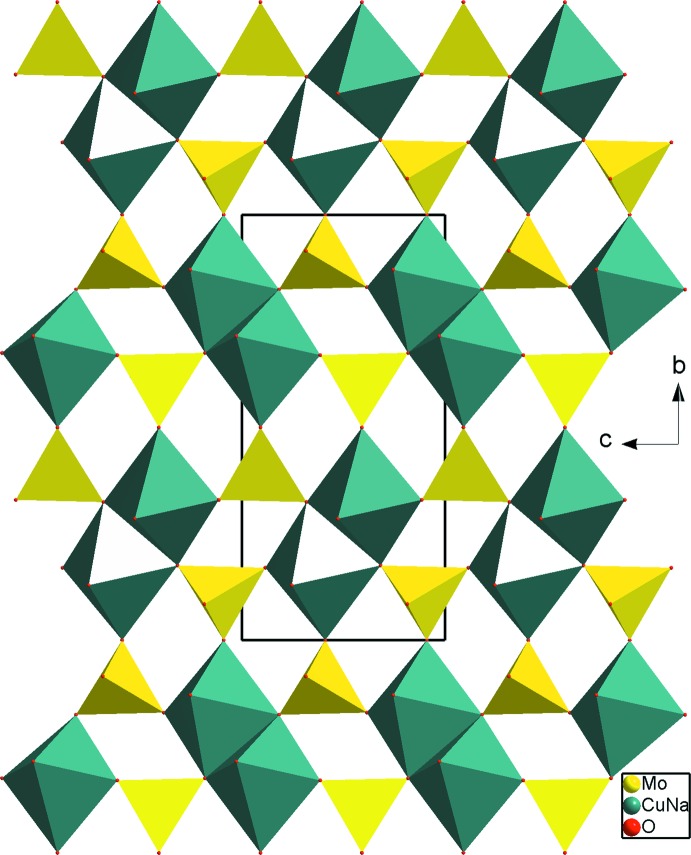
A projection of the polyhedral layers in the *bc* plane.

**Figure 3:**
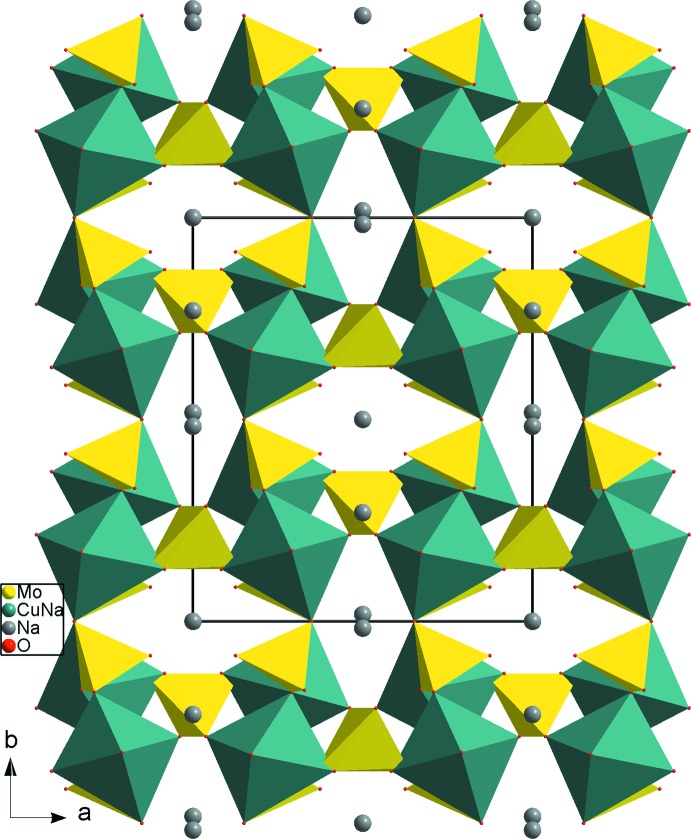
A projection of the β-Na_4_Cu(MoO_4_)_3_ structure, viewed normal to (001), showing the channels where monovalent cations are located.

**Figure 4:**
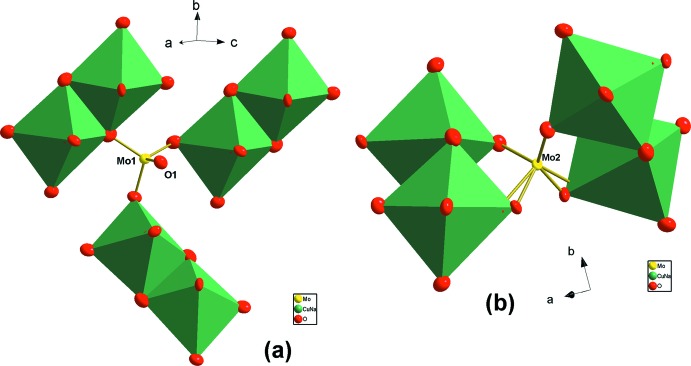
The association modes of *M*_2_O_10_-based units by the (*a*) Mo(1)O_4_ and (*b*) Mo(2)O_4_ tetra­hedra. For clarity, we present only one atom of molybdenum, Mo2.

**Figure 5:**
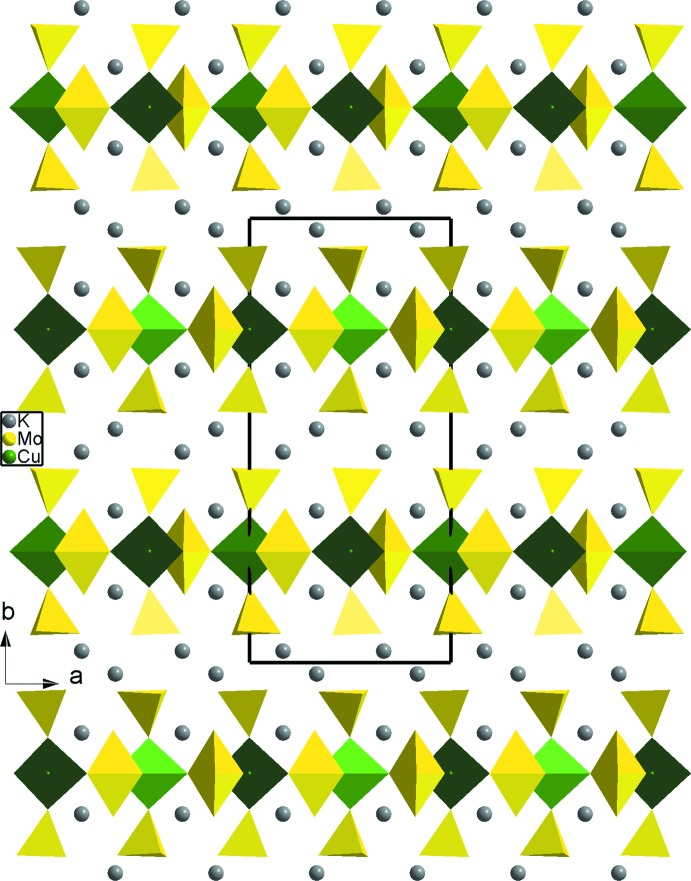
A projection of the K_4_Cu(MoO_4_)_3_ structure, viewed along the [001] direction.

**Figure 6:**
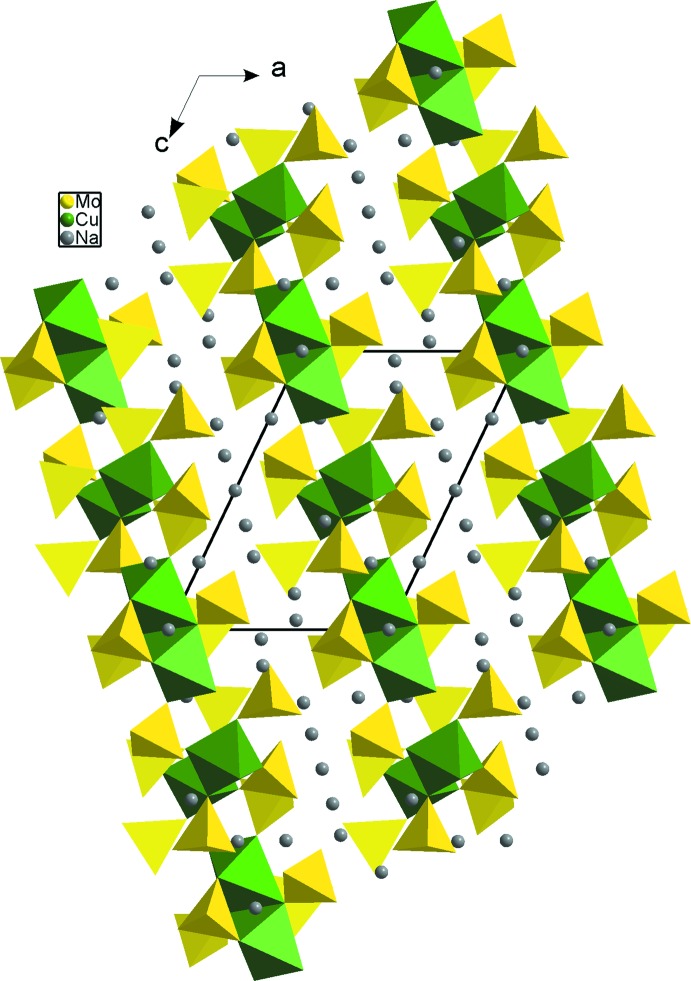
A projection of the α-Na_4_Cu(MoO_4_)_3_ structure, viewed in the (010) plane.

**Figure 7:**
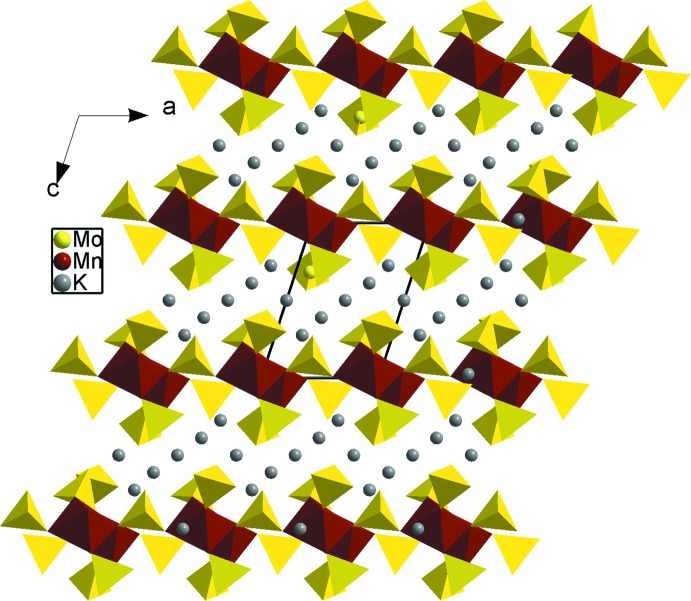
A projection of the K_4_Mn(MoO_4_)_3_ structure, viewed normal to (010).

The structural unit in β-Na_4_Cu(MoO_4_)_3_ is formed by *M*O_6_ (*M* = Cu1/Na1) octa­hedra linked by sharing vertices with Mo1O_4_ tetra­hedra and two slightly different Mo2O_4_ tetra­hedra, with a partially occupied (0.5 occupancy) Mo2 site. Atom O4 is split into two separate positions, with occupancies of 0.5 for the O4 and O41 atoms. The charge compensation is provided by Na^+^ cations (Fig. 1 ▸). The essential building units of the structure are *M*_2_O_10_ units obtained from two edge-sharing *M*O_6_ octa­hedra. These units are connected by Mo1O_4_ tetra­hedra through vertex-sharing *via* Mo—O—*M* mixed bridges. This results in *M*_2_Mo_2_O_16_ units. Each unit is connected to six other identical units by the sharing of vertices, leading to an infinite layer of the *M*_2_Mo_3_O_14_ type parallel to the (100) plane (Fig. 2 ▸). The linkage of these layers is ensured by the two slightly different Mo2O_4_ tetra­hedra, linking *via* corners. This results in a three-dimensional framework delimited by two kinds of channels running along the *c* axis at (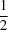, 0, *z*) and (0, 0, *z*). These channels are occupied by Na^+^ cations (Fig. 3 ▸). In the anionic framework, each Mo2O_4_ tetra­hedron shares its O atoms with four different *M*_2_O_10_ dimers belonging to two adjacent layers. The Mo1O_4_ tetra­hedron shares only three O atoms with three *M*_2_O_10_ units belonging to the same layer, the other O atom being free and pointing towards the channels where the Na3 cations are located (Fig. 4 ▸). There is some compositional flexibility in the alluaudite structure and the studied material is isostructural with Na_5_Sc(MoO_4_)_4_ (Klevtsova *et al.*, 1975 ▸) and Na_3_In_2_As_3_O_12_ (Khorari *et al.*, 1997 ▸). The two crystallographically independent Mo atoms have tetra­hedral coordination, with an average Mo—O distance of 1.761 Å for Mo1 and 1.777 Å for Mo2, which is in a good agreement with those typically observed in Rb_2_Cu_2_(MoO_4_)_3_ (Solodovnikov & Solodovnikova, 1997 ▸). The Na^+^ cations occupy three crystallographically independent sites with different O-atom environments. The Na2, Na3 and Na4 atoms are surrounded by four, eight and six O atoms, respectively (Table 1 ▸). The Cu1 and Na1 cations are located at the same general site, with occupancies of 0.5, and have an octa­hedral environment with an average distance of 2.214 Å. This distance presents a mean between the Na—O distances of Na_2_Cu(PO_3_)_4_ (Laügt *et al.*, 1972 ▸) and the Cu—O distances encountered in Ag_2_Cu_2_(MoO_4_)_3_ (Tsyrenova *et al.*, 2009 ▸). The proposed structural model is confirmed by two validation models: (i) the bond-valence approach using the empirical formula of Brown (Brown & Altermatt, 1985 ▸) and (ii) the charge-distribution method Chardi (Nespolo, 2015 ▸, 2016 ▸). The charge distribution method is the most recent development of Pauling’s concept of bond strength (Pauling, 1929 ▸). Instead of empirical parameters used in the bond-valence approach, it exploits the experimental bond lengths deduced from the structural study to compute a non-integer coordination number, ECoN (effective coordination number), around a PC-atom (atom placed at the center of a polyhedron, *q* > 0), which is coordinated by V atoms (atoms located at the vertices, *q* < 0); *q* is the formal oxidation number. ECoN takes into account not only the number of V atoms around a given PC atom, but also their weight in terms of relative distances. Calculated charges *Q*(i) and valences *V*(i) are in good agreement with the formal oxidation number (*q*) multiplied by occupancy rates. The dispersion factor MAPD, which measures the mean absolute percentage deviation, is 2.2% for the calculated cationic charges. The variation of the ECoN value to the traditional coordination indicates the degree of distortion. The two validation models results are summarized in Table 2 ▸. Comparing our structure with that of a similar formulation, *i.e.* K_4_Cu(MoO_4_)_3_ (Menard *et al.*, 2011 ▸), we found a clear difference, on the one hand, in the crystal symmetry and, on the other hand, in the arrangement of polyhedra. K_4_Cu(MoO_4_)_3_ crystallizes in the *Pnma* space group. Its structure can be described as being composed of a distorted square-planar CuO_4_ polyhedron bound by shared vertices to two Mo1O_4_ tetra­hedra to form CuMo_2_O_10_-type units. These units are inter­connected, on the one hand, by insertion of two Mo2O_4_ tetra­hedra which share a face with a partial occupation (0.5 occupancy) of Mo2 atoms, and secondly by forming a mixed bridge of the Mo—O—Cu type. This forms ribbons arranged parallel to the [100] direction. This results in a one-dimensional structure in which K^+^ atoms reside in the inter-ribbon spaces (Fig. 5 ▸). The structure of our new variety β-Na_4_Cu(MoO_4_)_3_ is compared with the α variety. Indeed, α-Na_4_Cu(MoO_4_)_3_ (Klevtsova *et al.*, 1991 ▸) crystallizes in the triclinic system, space group *P* 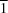, and its structure is formed by the same Cu_2_O_10_ dimers present in our structure (here present as mixed-occupied *M*_2_O_10_ units). The latter connects *via* Mo—O—Cu double composite bridges with two bidentate tetra­hedra MoO_4_ and by Mo—O—Cu simple bridges with monodentate MoO_4_ tetra­hedra to form Cu_2_Mo_4_O_20_ units. The Cu_2_Mo_4_O_20_ units are connected by MoO_4_ tetra­hedra and the pooling of vertices to form ribbons arranged in the [010] direction. All the ribbons form a one-dimensional framework with inter-ribbon spaces containing monovalent Na^+^ cations (Fig. 6 ▸). This structure has the same arrangement of structural units found in the one-dimensional structure of K_3_Mn(MoO_4_)_3_ (Solodovnikov *et al.*, 1998 ▸) (Fig. 7 ▸).

## Synthesis and crystallization   

β-Na_4_Cu(MoO_4_)_3_ crystals were obtained by a solid-state reaction from the following reagents: Na_2_CO_3_ (Prolabo, 70128), Cu(CO_2_CH_3_)·H_2_O (Sigma–Aldrich, C5893) and (NH_4_)_6_Mo_7_O_24_·4H_2_O (Sigma–Aldrich, 13301) with an Na:Cu:Mo molar ratio of 4:1:3. The resulting mixture was milled in an agate mortar, placed in a porcelain crucible and then preheated slowly in air at 623 K for 24 h, in order to eliminate volatile products. Thereafter, it was heated to a temperature close to that of the fusion at 873 K. It was left at this temperature for 20 d to induce nucleation and crystal growth. The final residue was first cooled slowly (5 K per half day) to 823 K and then rapidly (50 K h^−1^) to room temperature. Green crystals of sufficient size for the measurement of intensities were obtained.

## Refinement   

Crystal data, data collection and structure refinement details are summarized in Table 3 ▸. We used of EADP and EXYZ constraints within *SHELXL2014* (Sheldrick, 2015 ▸) for Cu1/Na1 located at the same crystallographic site. Atom O4 was split over two sites (O4 and O41) as this displayed a very elongated displacement ellipsoid. The occupancies of O4 and O41 were set to 0.5 in line with the occupany of Mo2; the two separate O-atom sites (O4 and O41) correspond to two different orientations of the Mo2O_4_ tetra­hedron related by symmetry. Refining atomic occupancies leads to a value of 0.497 (4) for the Cu atom. For conditions of electrical neutrality, we set the occupancy of the Cu atom as 0.5. This leads to well-defined ellipsoids. The maximum and minimum electron densities in the final Fourier difference map are acceptable and located at 0.77 and 0.82 Å, respectively, from the Na2 and Mo1 atoms.

## Supplementary Material

Crystal structure: contains datablock(s) I. DOI: 10.1107/S2056989016010367/pj2032sup1.cif

Structure factors: contains datablock(s) I. DOI: 10.1107/S2056989016010367/pj2032Isup2.hkl

CCDC reference: 1487800

Additional supporting information: crystallographic information; 3D view; checkCIF report

## Figures and Tables

**Table 1:** Selected bond lengths (Å)

Mo1—O1	1.746 (3)	Na2—O5^iv^	2.462 (3)
Mo1—O5	1.762 (3)	Na2—O5	2.549 (3)
Mo1—O2^i^	1.764 (3)	Na2—O5^ii^	2.549 (3)
Mo1—O6	1.774 (3)	Na3—O41^vii^	2.443 (7)
Mo2—Mo2^ii^	0.447 (2)	Na3—O41^viii^	2.443 (7)
Mo2—O41	1.456 (7)	Na3—O1^ix^	2.493 (3)
Mo2—O3^ii^	1.738 (3)	Na3—O1^ii^	2.493 (3)
Mo2—O41^ii^	1.740 (7)	Na3—O1^vi^	2.675 (3)
Mo2—O4	1.787 (7)	Na3—O1^x^	2.675 (3)
Mo2—O3	1.822 (3)	Na3—O4^vii^	2.749 (7)
Mo2—O4^ii^	2.150 (6)	Na3—O4^viii^	2.749 (7)
Cu1—O4^iii^	1.884 (6)	Na3—O41^xi^	3.008 (7)
Cu1—O3	2.098 (3)	Na3—O41^xii^	3.008 (7)
Cu1—O2	2.116 (3)	Na4—O3	2.337 (2)
Cu1—O6	2.152 (3)	Na4—O3^xiii^	2.337 (2)
Cu1—O5^iv^	2.317 (3)	Na4—O2	2.424 (3)
Cu1—O6^v^	2.464 (4)	Na4—O2^xiii^	2.424 (3)
Cu1—O41^iii^	2.467 (4)	Na4—O1^v^	2.490 (4)
Na2—O5^vi^	2.462 (3)	Na4—O1^xiv^	2.490 (4)

Symmetry codes: (i) 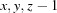; (ii) 
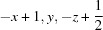
; (iii) 
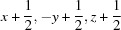
; (iv) 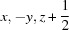; (v) 
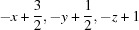
; (vi) 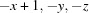; (vii) 
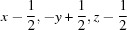
; (viii) 
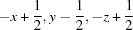
; (ix) 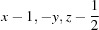; (x) 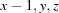; (xi) 
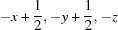
; (xii) 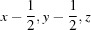; (xiii) 
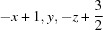
; (xiv) 
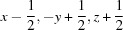
.

**Table 2:** CHARDI and BVS analysis of cation polyhedra in β-Na_4_Cu(MoO_4_)_3_

Cation	*q*(i)·sof(i)	*Q*(i)	V(i).sof(i)	CN(i)	ECoN(i)	*d*_ar_	*d*_med_
Mo1	6.00	6.24	5.93	4	4.00	1.761	1.761
Mo2	3.00	2.48	3.06	4	3.52	1.777	1.776
*M*	1.50	1.75	1.64	6	4.97	2.214	2.214
Na2	1.00	0.98	0.79	4	4.49	2.505	2.703
Na3	1.00	0.83	0.85	8	8.19	2.587	2.670
Na4	1.00	1.24	1.11	6	5.86	2.417	2.417

Notes: *M* = Cu1/Na1, *q*(i) = formal oxidation number, sof(i) = site-occupation factor, *Q*(i) = calculated charges, CN = coordination number, ECoN = number of effective coordination, MAPD = 100/NΣ_i_^N^.|*q*i - *Q*i/*q*i|, *d*_ar_ = arithmetic average distance and *d*_med_ = weighted average distance.

**Table 3:** Experimental details

Crystal data	
Chemical formula	Na_4_Cu(MoO_4_)_3_
*M*_r_	635.32
Crystal system, space group	Monoclinic, *C*2/*c*
Temperature (K)	298
*a*, *b*, *c* (Å)	12.5318 (9), 13.8181 (9), 7.1159 (7)
β (°)	111.95 (2)
*V* (Å^3^)	1142.9 (2)
*Z*	4
Radiation type	Mo *K*α
μ (mm^−1^)	5.26
Crystal size (mm)	0.28 × 0.22 × 0.18

Data collection	
Diffractometer	Enraf–Nonius CAD-4
Absorption correction	ψ scan (North *et al.*, 1968 ▸)
*T*_min_, *T*_max_	0.224, 0.387
No. of measured, independent and observed [*I* > 2σ(*I*)] reflections	2678, 1238, 1208
*R*_int_	0.030
(sin θ/λ)_max_ (Å^−1^)	0.638

Refinement	
*R*[*F* ^2^ > 2σ(*F* ^2^)], *wR*(*F* ^2^), *S*	0.023, 0.058, 1.17
No. of reflections	1238
No. of parameters	104
Δρ_max_, Δρ_min_ (e Å^−3^)	0.80, −0.72

Computer programs: *CAD-4 EXPRESS* (Duisenberg, 1992 ▸), *XCAD4* (Harms & Wocadlo, 1995 ▸), *SHELXS97* (Sheldrick, 2008 ▸), *SHELXL2014* (Sheldrick, 2015 ▸), *DIAMOND* (Brandenburg & Putz, 2001 ▸), *WinGX* (Farrugia, 2012 ▸) and *publCIF* (Westrip, 2010 ▸).

## supplementary crystallographic information

### Crystal data

**Table:**

Na_4_Cu(MoO_4_)_3_	*F*(000) = 1180
*M**_r_* = 635.32	*D*_x_ = 3.692 Mg m^−^^3^
Monoclinic, *C*2/*c*	Mo *K*α radiation, λ = 0.71073 Å
*a* = 12.5318 (9) Å	Cell parameters from 25 reflections
*b* = 13.8181 (9) Å	θ = 10–15°
*c* = 7.1159 (7) Å	µ = 5.26 mm^−^^1^
β = 111.95 (2)°	*T* = 298 K
*V* = 1142.9 (2) Å^3^	Prism, green
*Z* = 4	0.28 × 0.22 × 0.18 mm

### Data collection

**Table:**

Enraf–Nonius CAD-4 diffractometer	1208 reflections with *I* > 2σ(*I*)
ω/2θ scans	*R*_int_ = 0.030
Absorption correction: ψ scan (North *et al.*, 1968)	θ_max_ = 27.0°, θ_min_ = 2.3°
*T*_min_ = 0.224, *T*_max_ = 0.387	*h* = −15→15
2678 measured reflections	*k* = −1→17
1238 independent reflections	*l* = −9→9

### Refinement

**Table:**

Refinement on *F*^2^	0 restraints
Least-squares matrix: full	*w* = 1/[σ^2^(*F*_o_^2^) + (0.0173*P*)^2^ + 5.2182*P*] where *P* = (*F*_o_^2^ + 2*F*_c_^2^)/3
*R*[*F*^2^ > 2σ(*F*^2^)] = 0.023	(Δ/σ)_max_ = 0.001
*wR*(*F*^2^) = 0.058	Δρ_max_ = 0.80 e Å^−^^3^
*S* = 1.17	Δρ_min_ = −0.72 e Å^−^^3^
1238 reflections	Extinction correction: SHELXL2014 (Sheldrick, 2015), Fc^*^=kFc[1+0.001xFc^2^λ^3^/sin(2θ)]^-1/4^
104 parameters	Extinction coefficient: 0.00081 (11)

### Special details

**Table:**

Geometry. All esds (except the esd in the dihedral angle between two l.s. planes) are estimated using the full covariance matrix. The cell esds are taken into account individually in the estimation of esds in distances, angles and torsion angles; correlations between esds in cell parameters are only used when they are defined by crystal symmetry. An approximate (isotropic) treatment of cell esds is used for estimating esds involving l.s. planes.

### Fractional atomic coordinates and isotropic or equivalent isotropic displacement parameters (Å^2^)

**Table:**

	*x*	*y*	*z*	*U*_iso_*/*U*_eq_	Occ. (<1)
Mo1	0.72812 (2)	0.10879 (2)	0.11920 (4)	0.01991 (12)	
Mo2	0.48085 (7)	0.28610 (4)	0.2400 (3)	0.0133 (3)	0.5
Cu1	0.70643 (5)	0.16100 (5)	0.61691 (9)	0.01741 (16)	0.5
Na1	0.70643 (5)	0.16100 (5)	0.61691 (9)	0.01741 (16)	0.5
Na2	0.5000	0.0169 (3)	0.2500	0.0482 (8)	
Na3	0.0000	0.0000	0.0000	0.0299 (5)	
Na4	0.5000	0.26934 (16)	0.7500	0.0219 (4)	
O1	0.8745 (2)	0.0849 (2)	0.1859 (4)	0.0292 (6)	
O2	0.6715 (2)	0.1702 (2)	0.8852 (4)	0.0337 (6)	
O3	0.5390 (2)	0.2147 (2)	0.4714 (4)	0.0247 (6)	
O4	0.3657 (5)	0.3629 (6)	0.2376 (11)	0.0239 (10)	0.5
O41	0.4153 (5)	0.3707 (6)	0.2551 (11)	0.0239 (10)	0.5
O5	0.6504 (3)	−0.0001 (2)	0.0904 (5)	0.0346 (7)	
O6	0.7095 (3)	0.1754 (2)	0.3178 (5)	0.0373 (7)	

### Atomic displacement parameters (Å^2^)

**Table:**

	*U*^11^	*U*^22^	*U*^33^	*U*^12^	*U*^13^	*U*^23^
Mo1	0.01593 (17)	0.02069 (18)	0.01973 (18)	0.00034 (10)	0.00275 (11)	−0.00104 (11)
Mo2	0.0147 (8)	0.0162 (2)	0.0079 (4)	0.0004 (2)	0.0032 (6)	0.0000 (3)
Cu1	0.0227 (3)	0.0219 (3)	0.0093 (3)	−0.0007 (3)	0.0079 (2)	0.0006 (2)
Na1	0.0227 (3)	0.0219 (3)	0.0093 (3)	−0.0007 (3)	0.0079 (2)	0.0006 (2)
Na2	0.0217 (11)	0.098 (3)	0.0203 (11)	0.000	0.0031 (9)	0.000
Na3	0.0397 (12)	0.0224 (10)	0.0179 (9)	−0.0013 (10)	−0.0004 (9)	−0.0006 (9)
Na4	0.0225 (9)	0.0270 (11)	0.0189 (9)	0.000	0.0110 (7)	0.000
O1	0.0187 (12)	0.0341 (15)	0.0307 (14)	0.0037 (11)	0.0044 (10)	0.0072 (12)
O2	0.0268 (14)	0.0330 (16)	0.0327 (15)	0.0083 (12)	0.0011 (12)	0.0017 (13)
O3	0.0275 (13)	0.0335 (15)	0.0160 (12)	−0.0003 (12)	0.0113 (10)	0.0011 (11)
O4	0.017 (3)	0.0250 (18)	0.0238 (18)	0.005 (3)	0.001 (3)	−0.0039 (16)
O41	0.017 (3)	0.0250 (18)	0.0238 (18)	0.005 (3)	0.001 (3)	−0.0039 (16)
O5	0.0357 (15)	0.0279 (14)	0.0410 (17)	−0.0059 (13)	0.0152 (13)	0.0005 (13)
O6	0.0305 (14)	0.0398 (17)	0.0385 (17)	0.0010 (14)	0.0095 (13)	−0.0084 (14)

### Geometric parameters (Å, º)

**Table:**

Mo1—O1	1.746 (3)	Na2—O5^iv^	2.462 (3)
Mo1—O5	1.762 (3)	Na2—O5	2.549 (3)
Mo1—O2^i^	1.764 (3)	Na2—O5^ii^	2.549 (3)
Mo1—O6	1.774 (3)	Na3—O41^vii^	2.443 (7)
Mo2—Mo2^ii^	0.447 (2)	Na3—O41^viii^	2.443 (7)
Mo2—O41	1.456 (7)	Na3—O1^ix^	2.493 (3)
Mo2—O3^ii^	1.738 (3)	Na3—O1^ii^	2.493 (3)
Mo2—O41^ii^	1.740 (7)	Na3—O1^vi^	2.675 (3)
Mo2—O4	1.787 (7)	Na3—O1^x^	2.675 (3)
Mo2—O3	1.822 (3)	Na3—O4^vii^	2.749 (7)
Mo2—O4^ii^	2.150 (6)	Na3—O4^viii^	2.749 (7)
Cu1—O4^iii^	1.884 (6)	Na3—O41^xi^	3.008 (7)
Cu1—O3	2.098 (3)	Na3—O41^xii^	3.008 (7)
Cu1—O2	2.116 (3)	Na4—O3	2.337 (2)
Cu1—O6	2.152 (3)	Na4—O3^xiii^	2.337 (2)
Cu1—O5^iv^	2.317 (3)	Na4—O2	2.424 (3)
Cu1—O6^v^	2.464 (4)	Na4—O2^xiii^	2.424 (3)
Cu1—O41^iii^	2.467 (4)	Na4—O1^v^	2.490 (4)
Na2—O5^vi^	2.462 (3)	Na4—O1^xiv^	2.490 (4)
			
O1—Mo1—O5	110.42 (15)	O3—Cu1—O2	85.28 (10)
O1—Mo1—O2^i^	111.01 (14)	O4^iii^—Cu1—O6	93.4 (2)
O5—Mo1—O2^i^	106.95 (15)	O3—Cu1—O6	82.16 (11)
O1—Mo1—O6	108.63 (14)	O2—Cu1—O6	166.61 (12)
O5—Mo1—O6	107.69 (15)	O4^iii^—Cu1—O5^iv^	96.1 (2)
O2^i^—Mo1—O6	112.08 (15)	O3—Cu1—O5^iv^	94.73 (11)
O3^ii^—Mo2—O4	118.4 (2)	O2—Cu1—O5^iv^	88.43 (12)
O3^ii^—Mo2—O3	110.75 (17)	O6—Cu1—O5^iv^	97.18 (12)
O4—Mo2—O3	112.3 (3)	O6^v^—Cu1—O2	89.55 (14)
O3^ii^—Mo2—O4^ii^	100.3 (2)	O6^v^—Cu1—O4^iii^	76.78 (15)
O4—Mo2—O4^ii^	113.9 (4)	O6^v^—Cu1—O5^iv^	172.13 (15)
O3—Mo2—O4^ii^	99.0 (2)	O6^v^—Cu1—O6	86.42 (14)
O4^iii^—Cu1—O3	168.8 (2)	O6^v^—Cu1—O3	92.66 (12)
O4^iii^—Cu1—O2	98.1 (2)		

Symmetry codes: (i) *x*, *y*, *z*−1; (ii) −*x*+1, *y*, −*z*+1/2; (iii) *x*+1/2, −*y*+1/2, *z*+1/2; (iv) *x*, −*y*, *z*+1/2; (v) −*x*+3/2, −*y*+1/2, −*z*+1; (vi) −*x*+1, −*y*, −*z*; (vii) *x*−1/2, −*y*+1/2, *z*−1/2; (viii) −*x*+1/2, *y*−1/2, −*z*+1/2; (ix) *x*−1, −*y*, *z*−1/2; (x) *x*−1, *y*, *z*; (xi) −*x*+1/2, −*y*+1/2, −*z*; (xii) *x*−1/2, *y*−1/2, *z*; (xiii) −*x*+1, *y*, −*z*+3/2; (xiv) *x*−1/2, −*y*+1/2, *z*+1/2.
